# Health of (dual) health professional students in German-speaking countries: a scoping review

**DOI:** 10.3389/fpubh.2023.1243324

**Published:** 2023-09-19

**Authors:** Ivonne-Nadine Jürgensen, Peter Koch, Annike Morgane Nock, Corinna Petersen-Ewert

**Affiliations:** ^1^Department of Nursing and Management, Faculty of Business and Social Science, University of Applied Sciences Hamburg, Hamburg, Germany; ^2^Competence Center for Epidemiology and Health Services Research for Healthcare Professionals (CVcare), Institute for Health Services Research in Dermatology and Nursing (IVDP), University Medical Center Hamburg-Eppendorf (UKE), Hamburg, Germany

**Keywords:** students, health occupations, health, German-speaking countries, university, health promotion

## Abstract

University education marks a new stage in life, which is associated with unknown demands and challenges and can have a negative impact on students’ health. Therefore, health promotion in the university setting is becoming increasingly important. In this context, scientific data on the health situation play a crucial role in improving students’ health. Thus, the aim of the scoping review was to highlight the current scope of research on the health of health professional students. It also explored problems and outlined key future challenges and solutions. The review was conducted using the Joanna Briggs Institute (JBI) methodology for a scoping review. A total of nine databases (PubMed, CINAHL, CareLit, LIVIVO, Scopus, Psyndex, PEDro, OTseeker, Google Scholar) were systematically searched. The following search criteria were defined: health professional students, health, Germany, German-speaking countries, all types of sources from 2012 to present are selected. The research studies were mapped in a table and health evidence of included studies was summarized narratively. The initial search resulted in 23,938 records. Seven records met the inclusion criteria and were included in the review. Six cross-sectional studies were conducted in Germany, and one cross-sectional study was conducted in Switzerland. In fact, one study included a representative population. Qualitative studies were not found. The most studies investigated health status, health behavior, and personal resources. Most of the studies examined female nursing students. The included studies indicated that the young students reported physical or mental health conditions. In addition, the studies also identified health resources of the students that need to be improved. In summary, there is currently limited health evidence on this group of students in German-speaking countries. Therefore, further research is needed to generate knowledge and comprehensively describe the health situation.

## 1. Introduction

### Rationale: healthcare industry in Germany

The healthcare industry is an important economic sector in Germany because it makes a crucial contribution to economic development and employment. Last year, the gross value added of this sector amounted to 439.6 billion Euros, accounting for 12.7% of the total gross value added of the German economy. In 2022, around 8.1 million people were employed in the healthcare industry (1). The healthcare industry consists of three main areas: Research and development, digitalization, and healthcare services. Healthcare services include outpatient and inpatient care for the population (2). From it results that, the healthcare sector offers diverse opportunities for growth, innovation, and employment. Furthermore, the population’s growing health awareness is leading to an overall increase in demand for professional healthcare services (1, 3). However, health services face various challenges, such as demographic change or a lack of qualified professionals to cope with the multiple opportunities and growing demands for healthcare (2). In addition, Germany has become a country of immigration. The healthcare sector therefore is faced with the task of addressing the healthcare needs of migrants (4). The nursing profession is a good example of this. Nurses represent the largest occupational group in the German healthcare sector (5). The global COVID-19 pandemic has highlighted the importance of nursing profession for the healthcare of the entire population (6, 7). This profession is particularly affected by demographic change and a lack of qualified professionals. The current shortage of personnel cannot be compensated by enough young professionals (8, 9). Considering this, it is important to have an adequate offer of qualified and health professionals. These challenges not only represent risk for economic development but also emphasize one of the most important socio-political tasks in the coming years in Germany (1, 3).

### Health professions in Germany–new demands and fields of action

Health professionals are already facing with many new demands and tasks in relation to the healthcare of the population. These increasing demands are a result of the rising life expectancy and the increase in the proportion of people with multiple chronic illnesses (9) such as dementia patients (10). Moreover, there are special demands on the healthcare of people with a migration background (4). To meet these demands and tasks, expanded skills and (intercultural) competencies are necessary to ensure high-quality and interdisciplinary healthcare in the future (4, 8). Furthermore, the professional field of activity in the healthcare sector is expanding, for example, in the context of health promotion (11) as well as in promoting patients’ health literacy (12).

### Professionalization of health professions in Germany

To meet these demands, the professionalization of health professions is one approach (13). In 2012, the German Science Council recommended that a significant proportion (10–20%) of health professionals should receive university education (13). The Council also recommends that experienced health professionals are offered the opportunity for further academic qualification. For university education, a primary qualifying bachelor’s degree program with a patient-oriented focus is recommended, which prepares students directly for working with patients (13). In this context, the Council considers a dual study program to be a suitable form of education (14). The Nursing Professions Act (2020) validates a primary dual bachelor’s degree program at universities. This law represents an important step toward upgrading the nursing profession, providing international career opportunities, and addresses new target groups to enter the profession (15).

### Bologna-reform

Students are meeting changed study conditions due to the implementation of the Bologna reform. The Bologna reform has led to a stronger structuring of bachelor’s programs, which is evident in their modular organization (16). Dual bachelor’s degree programs are highly structured as they include two educational settings: the university and practical experience (17). Nevertheless, there is an increasing demand for dual degree programs in the healthcare sector (18).

### Study time

The start of studying highlights a new phase of life, which is associated with challenges for young adults (19) and for experienced health professionals (20). During this life changes, they face new demands and difficulties. Students, for example, are exposed to different stressors, e.g., academic workload, learning and time management struggles, uncertainties, high frequency of exams (19, 21). Coping with these demands self-competencies, are independence and a sense of responsibility are needed (19, 21). In addition, the study phase represents a life stage for young people also connected with transition to adulthood (22). Arnett (2000) defined this life phase between 18 and 25 years as “emerging adulthood” and pointed out that risky lifestyles are most practiced in this phase (23).

### Students health during study time

In the past, students were considered as a group with few health issues because of their young age (24). However, with the implementation of the Bologna reform and its impact as well as COVID-19 pandemic, student health has become a significant research topic in Germany (25–27). Evidence points to the fact that the pressure to academic perform and study-related stress can have a negative influence on students’ subjective health (25, 28, 29). A nationwide report on student’s health in Germany shows that students have a poorer self-assessment of their subjective health compared to employed people of the same age and suffer more frequently from mental burdens (30). Undergraduate students experience the highest levels of subjective stress, which can result in sleep problems and impaired sleep quality (31, 32). Research from the English-speaking countries shows that students in health-related degree programs are exposed to particularly high levels of stress, due to the academic workload and practical training (33, 34). In this regard, academic workload and clinical practice assignments are specific stressors. In their clinical practice, they are frequently confronted with patients suffering, illness, and death. Students must learn how to deal with the conditions of practice to perform in a professional way (33, 35). Another significant stress factor in practice is that the academic qualification is not yet fully accepted in Germany and a critical attitude toward students is prevalent (36).

### Health promotion in the university setting

According to the World Health Organization (WHO), universities offer enormous possibilities for health promotion, which can have a positive impact on student’s subjective health, well-being, and their academic success (37) and contributes to reduce health disparities (26). In summary, completing a degree program relates to demands that can influence health. Students represent a relevant group for health promotion, which should not be ignored in the university setting. Therefore, the present scoping review highlights the current scope of research regarding the health of health professional students in German-speaking countries. It also explores problems and outline key future challenges and solutions. In addition, the scoping review provide a solid basis for future research initiatives.

### Objectives

This scoping review aimed to (i) identify the scope of existing research on health of health professional students in Germany or German-speaking countries and (ii) provide an overview of the findings. The research objectives were operationalized using two research questions.

*Which empirical studies have been conducted on the health of health professional students in German-speaking countries?*

*What has been reported on health outcomes of health professional students in German-speaking countries?*

## 2. Materials and methods

For this scoping review, we used the Joanna Briggs Institute (JBI) methodology for scoping review (38). According to the JBI methodology (38), a preliminary protocol based on the JBI guidelines (39) was published in December 2022 (40).

The inclusion criteria based on the PCC framework. PCC means population, concept, and context (38). Inclusion criteria: *Population*: health professional students (nursing, physiotherapy, occupational therapy, speech therapy). *Concept*: health as a multidimensional concept (measured by scientific health indicators). *Context:* official German-speaking countries (Germany, Switzerland, Austria), publication date: from 2012 to present (here, the science council made its recommendation for professionalization). We left the source of information “open.” Thus, we included all existing types of information sources and study designs. A total of nine databases were screened: MEDLINE (PubMed), CINAHL (EBESCO), CareLit (German-language journals), LIVIO, Scopus, Psyndex, PEDro, OTseeker, OpenGrey Library (University of London) and Google Scholar. The online catalog of the university library was also screened.

The search strategy included three steps. In the first step, we conducted an initial limited search (September 4, 2022) in MEDLINE (PubMed), using suitable keywords and Medical Subject Headings (MeSH) for “health profession* student*,” health, “German-speaking area,” limited by abstract, in the last 10 years, humans, English, German, adults aged 19+ years (40). Keywords and index terms in title and abstract were analyzed and used to refine the following inclusive search. Consultation with an experienced librarian at the university was helpful in designing and refining the electronic database search. We started our inclusive search, without claim of completeness, on December 15, 2022. The first author (INJ) screened every record by title for inclusion criteria and duplicates. A second search was performed using all identified keywords. In databases, we combined the defined keywords with the Boolean operators OR/AND. For example, the search term in MEDLINE (PubMed): #1 (′′Students, Health Occupations′′ [Mesh] OR ′′health profession* student*′′ OR ′′healthcare student*′′ OR ′′health-care student*′′ OR ′′health care student*′′ OR “academic health profession*” OR “dual studie*” OR “nursing student*” OR “physiotherapy student*” OR “physical therapy student*” OR “occupational therapy* student*” OR “speech therapy student*” OR “allied health student*”) #2 AND (health OR healthy) #3 AND (“subjective health status” OR “health-related quality of life” OR “quality of life” OR “well-being” OR “health perception” OR “perceived health” OR “health-related lifestyle factors” OR “health problem*” OR “health promotion” OR “physical health” OR “physical health problem*” OR “physical inactivity” OR “physical activity” OR “mental well-being” OR “mental health” OR “mental health problem*” OR “tobacco use” OR “substance* use” OR smok* OR cigarette OR alcohol OR “alcohol consumption” OR drug OR “eating behavio*” OR “nutritional habit*” OR “body mass index” OR “body weight” OR overweight OR obese OR “health-related behavio*” OR “risk health behavio*” OR “health behavior” OR “unhealthy behavio*” OR medication OR “health literacy” OR “self-efficacy” OR stress* OR burden OR “stress level” OR “academic requirements”) #4 AND (German* OR “german*-speaking region” OR “german*-speaking area” OR switz* OR Austria) #5 #1 AND #2 AND #3 AND #4 #6 #1 AND #2 AND #3 AND #4 (Filters: Abstract, last 10 years, Humans, English, German, Adult: 19+ years; Literature search performed: February 20, 2023). For the other databases, the search term was adapted to the respective options of another database (Appendix 1). In the third step, reference lists of included articles were checked for additional sources. The complete electronic search strategy is summarized in Appendix 1. The search was finalized on April 20, 2023.

The matched records were evaluated in EndNote (literature management software) for a second title/abstract screen, performed by two authors (INJ/AMN) based on the inclusion criteria. After title and abstract screening, *n* = 25 records were removed from EndNote. Thereafter, *n* = 17 full-text articles were assessed for eligibility. Suitable full-text articles (*n* = 14) were new grouped in EndNote and reviewed by one author (INJ). An additional, less systematic search was conducted in the reference lists of full-text articles. Any discrepancies in source selection were resolved through consensus and discussion with another reviewer (AMN). Relevant data from included articles were extracted using a table as stated in the priori-protocol (40). The table was based on the inclusion criteria and review questions. The data collection form was assessed by two authors (INJ/AMN) prior to data extraction. One author (INJ) read and extracted relevant data from the included records (*n* = 7) in line with the research questions. Evidence was summarized in table and described descriptively. Two authors (AMN/CPE) reviewed the final table for accuracy.

## 3. Results

### Results of search

**Figure 1:**
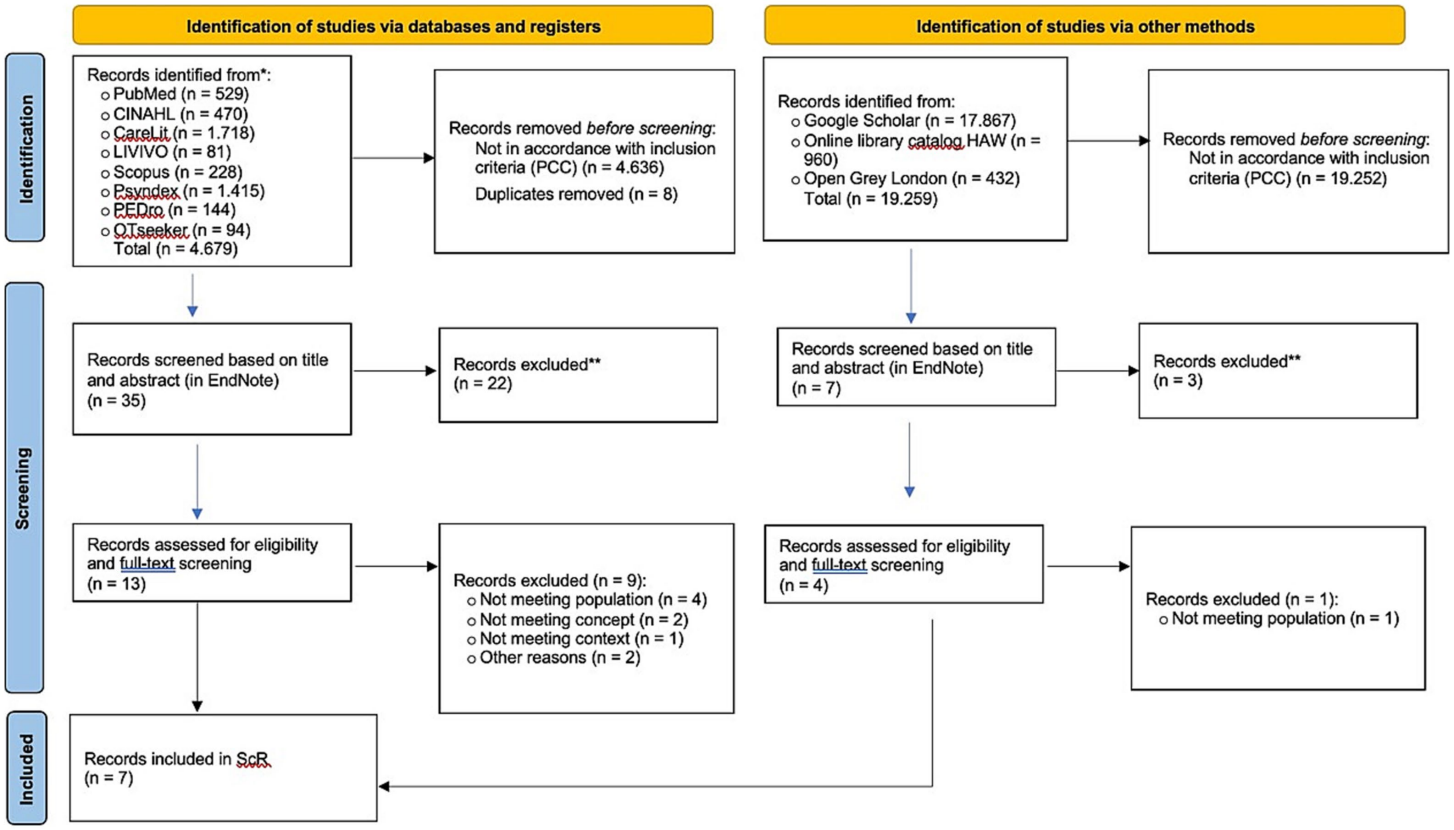
PRISMA flow diagram (41). For more information, visit: http://www.prisma-statement.org/.

The initial search resulted in 23.938 records (*n* = 4.679 records by searching electronic databases; *n* = 19.259 records by searching Google Scholar and online library catalogs). Of these, *n* = 23.896 records were excluded after title screening, and eight duplicates were removed. After excluded, *n* = 42 records were considered for a second screening in EndNote. Twenty-five records were excluded. In conclusion, 17 articles were assessed for eligibility. As a result, seven articles met our inclusion criteria. The flowchart (Figure 1) provides transparent documentation of record selection and exclusion process.

#### Results of first research question: which empirical studies have been conducted on the health of health professional students in German-speaking countries?

We identified seven articles (42–48). The articles addressing health of health professional students using quantitative study designs. Nursing students were most frequently surveyed regarding their health. The studies were published between 2014 and 2021. Six cross-sectional studies were conducted in Germany (GER). One cross-sectional study was performed in Switzerland (CH), which was based on a representative population with a comparative secondary analysis design (42). The studies examined key health dimensions, including health status, health behaviors, and personal resources. A total of five of the seven studies assessed personal resources. Self-efficacy, health literacy, and resilience were the three main dimensions of research interest. Three studies investigated students’ subjective physical and mental health status. One study (43) examined aspects of individual health behavior during the study period (Table 1). Qualitative studies were not found. Table 1 provides a brief overview of the included research articles addressing the health of health professional students.

**Table 1:** A brief overview of identified and included articles (cross-sectional studies; *n* = 7).

Author/year	Population	Concept	Context
Crawford et al. (2018) (42)	Nursing students etc.	Physical health status (back health)	CH
Hennersdorf and Schmidt (2019) (43)	Nursing students	Subjective physical and mental health status, health conditions, health behavior indicators (e.g., smoking, sleep, eating habits)	GER
Hermann et al. (2015) (44)	Nursing students (dual)	Personal resources: self-efficacy	GER
Rath and Lehmann (2020) (45)	Speech therapy students	Personal resources: resilience	GER
Reichardt and Petersen-Ewert (2014) (46)	Nursing students (dual)	Health-related quality of life (physical and mental), Personal resources: self-efficacy	GER
Reick and Hering (2018) (47)	Health professional students	Personal resources: health literacy	GER
Simon et al. (2021) (48)	Nursing students (dual) etc.	Personal resources: health literacy	GER

#### Results of second research question: what has been reported on health outcomes of health professional students in German-speaking countries?

The included articles reported different health outcomes.

##### Health status

The findings in the study by Crawford et al. showed a worryingly high prevalence for back pain and neck pain in young health professional students compared to the general Swiss population (42). The highest prevalence of neck pain was found in midwifery students (83%), and the lowest prevalence in nursing students (72%) (42) (Table 2). The authors concluded, “*These results are particularly concerning for a group yet to embark on their careers in professions that may be deemed more physically hazardous than for many other professions*” (42), p. 8. In terms of students’ subjective health status, some variations were evidenced (43). More than half of the students (51%) reported general health problems. The most common health problems were back pain (52%), followed by headache (28%) and fatigue (24%) (43). Subjective stress levels were reported as “very stressed” by 22% of the students. The compatibility of study and work was rated as “very difficult” by 61% of the students. A total of 31% of the participants stated that their subjective health had significantly worsened due to stressed and busy lifestyle (43) (Table 2). Hennersdorf and Schmidt discussed in their study that lack of time and stress seems to be important factors influencing the health of part-time students. Thus, the authors advised more focus on the health needs of health professional students (43). Furthermore, nursing students tended to show higher physical quality of life, but lower mental quality of life compared to a norm data group (46) (Table 2). The physical health scale score was 53.24 (SD: 5.49) and the mental health scale score was 45.24 (SD: 6.21). Scores range from 0 to 100, with higher scores indicating better physical and mental health (49). Reichardt and Petersen-Ewert (2014) concluded that students might be under mental stress at the beginning of their studies (46).

**Table 2:** Overview of the current state of health knowledge of health professional students.

Author/year	Population	Concept/study aims	Health outcomes/key findings
Crawford et al. (2018) (42)	Nursing students etc., Survey time: final study-year, *N* = 1848 Mean age: 25 Gender: 88% female	Examined prevalence of low back pain (LBP) and neck pain (NP), comparison to the Swiss population, Examined inter-professional differences in prevalence	Four-week prevalence LBP, all students: 61%; four-week prevalence LBP, in general Swiss population: 40% Four-week prevalence NP was higher in students (59%) than in the general Swiss population (36%) Yearly (crude) prevalence of LBP: 75% in the total student’s sample Highest yearly prevalence of LBP in midwifery students (81%), nursing students (77%), occupational therapy students (77%) Yearly (crude) prevalence for NP among all students: 74% Highest prevalence of NP was found in midwifery students (83%), nutritional sciences students (76%), occupational therapy students (75%), and nursing students (72%)
Hennersdorf and Schmidt (2019) (43)	Nursing students *N* = 30 (B.A.) *N* = 20 (M.A.) Mean age: 32.3 Gender: *n* = 39 female	Examined subjective health status	State of health: 10% “very good,” 49% “good,” 6% “bad” Self-reported health problems: back pain (52%), headaches (28%) and fatigue (24%) Stress perception: 22% “very stressed,” 52% as “rather stressed” Time to relax: 60% students had no time to relax. Studying and working: For nearly 61% very difficult. 31% students reported a worsened health due to time-stress and hectic. Subjective resource: For 51% students is the degree-program a subjective resource. Health situation during degree-program: 65% students reported that the subjective health situation did not change during their studies.
		Examined aspects of individual health behavior	Sleep behavior: 7 h per night Satisfied with own sleeping habit: every second student. Smoking: 76% non-smoker, 24% smoker Smoking habit during degree-program: 42% from the smoker reported a consistent smoking behavior. Drugs use: 98% never takes drugs. Painkiller tablet use: half of the students use occasionally. Eating habit: students reported a self-assessed unhealthy eating behavior during university time
Hermann et al. (2015) (44)	Nursing students (dual) Survey time: Semester 1, 3, 5, 7 *N* = 80 Gender: female: 62, male: 18	Investigated self-efficacy and the influence of the degree-program in the process of developing self-efficacy	Self-efficacy: Scale score total: 28.66 (SD: 3.85) Scale scores by semester: Semester 1: 29.07 (SD: 4.30), Semester 3: 24.47 (SD: 3.38), Semester 5: 29.28 (SD: 3.91) (the highest), Semester 7: 27.61 (SD: 3.58) Scale score by gender: f: 28.24 (SD: 3.60), m: 30.11 (SD: 4.42) Difference among semester and gender: no significant differences (semester: *p* = 0.55, gender: *p* = 0.07) No influence/correlation between degree-program and self-efficacy
Rath and Lehmann (2020) (45)	Speech therapy students *N* = 66 Survey time: Semester 2, 4, 6 Age: 18–34	Examined students’ resilience and related factors, e.g., depending on the semester, and indicated existing needs for resilience promotion	Resilience-Scale Score: Total 65 (SD: 10.8) Resilience-Scale Score by Semester: Semester 2: 64 (SD: 10.7), Semester 4: 64 (SD: 8.6), Semester 6: 68 (SD: 12.5) Differences between the students in resilience scores not statistically significant Resilience Score and age: slightly positive correlation (*r* = 0.02), but not significant (*p* = 0.85) Resilience and Well-being: positive correlation between resilience-score and well-being (*r* = 0.36, *p* = < 0.01), but a small, explained variance (*R*^2^ = 0.13).
Reichardt and Petersen-Ewert (2014) (46)	Nursing students (dual) *n* = 111 Age: 20.97 compared to: Nursing students (further education) (*n* = 73) and nursing apprentices (*n* = 52).	Investigated the health-related quality of life and self-efficacy	Physical health scale score: 53.24 (SD: 5.49) Mental health scale score: 45.24 (SD: 6.21) ➔ no significant differences between the three subsamples, neither in the physical nor in the mental summary scale Compared with a norm data group: Students have higher physical quality of life, but lower mental quality of life (strong effect *d* = 0.90). Self-efficacy scale score: 30.70 (SD: 3.43) Compared to norm data group: 29.43 (SD: 5.36) Students’ self-efficacy scale score is slightly higher as norm group
Reick and Hering (2018) (47)	Health professional students *N* = 127 *n* = 92 (health Department) Mean age: 24.1	Described students’ health literacy, and examined the influence of age, gender, and course-related factors on health literacy	Health literacy scale score: 31.1 (SD: 6.4) health professional students had problematic HL no significant differences in HL regarding gender, age, length of study, or department
Simon et al. (2021) (48)	Nursing students (dual) etc. *N* = 503 Mean age: 23.5 Gender: 83% female	Assessed students’ health literacy	Health literacy categories: 30% had a sufficient HL; almost 70% had a problematic or inadequate HL students with sufficient HL had a better subjective health status no sig. Differences in health literacy to gender or study program

##### Health behavior

Some students also reported unhealthy health behaviors, such as smoking or unhealthy eating habits during study time (43) (Table 2).

##### Personal resources self-efficacy

Hermann et al. (44) reported no significant differences (*p* = 0.55) in self-efficacy between their study samples (first semester: 29.07, SD: 4.30; third semester: 24.47, SD: 3.38; fifth semester: 29.28, SD: 3.91; seventh semester: 27.61, SD: 3.58). Furthermore, the authors found no significant difference (*p* = 0.07) regarding self-efficacy between genders (f: 28.24, SD: 3.60; m: 30.11, SD: 4.42). In terms of semesters, the fifth semester had the highest self-perceived self-efficacy. Hermann et al. (44) attributed this to the fact that the age of the students was not collected, although it has been described in the literature that self-efficacy ratings decrease with age (44). In addition, they found no response to the impact of the dual nursing degree program on the development of self-efficacy. Further research is recommended (44) (Table 2). Reichardt and Petersen-Ewert (2014) (46) also assessed self-efficacy in the student’s sample. The self-efficacy scale score in the study sample was slightly higher compared to a norm data group (30.70, SD: 3.34 vs. 29.43, SD: 5.36; Table 2). According to Schwarzer and Jerusalem (1999) (50), the scale ranges from 10 to 40, with a higher score indicating better subjective self-efficacy. Reichardt and Petersen-Ewert discussed that students had higher self-efficacy because they may have started the new stage of life with confidence (46).

##### Personal resources resilience

The overall resilience score among the assessed students ranged from 33 to 87, with an average score of 65 (SD: 10.8) (45). In accordance with Leppert et al. (51), there are three resilience categories: Score 13–66 “low resilience,” Score 67–72 “medium resilience,” Score 73–91 “high resilience.” Accordingly, most students in the three subsamples had low resilience scores. Participants in semesters two and four had the lowest mean resilience score (64, SD: 10.7; 64, SD: 8.6). The differences between students in resilience scores were not statistically significant (45) (Table 2). The research findings provide evidence that resilience should be promoted in students (45). To verify these findings, further surveys in additional study settings seem useful. In addition, ways to promote resilience should be tested and reflected in the curricula of these and other health professions (45).

##### Personal resources health literacy

In terms of health literacy, the results showed that health professional students had a problematic health literacy (47, 48). In the included studies, there were no significant differences in the level of health literacy with respect to gender, degree program (48), or completion of training in a health profession (47).

## 4. Discussion

### Summary of evidence

The objectives of this scoping review were to identify and describe existing (subjective) health related data from health professional students in Germany or in German-speaking countries. According to our state of knowledge this is the first scoping review that addresses and highlights the general body of evidence on the health of prospective academic professionals in Germany. The most important finding of this scoping review is that the research and data on health of this student group in Germany is not yet very extensive, although the topic has gained relevance in recent years (25, 26). A lack of data means not that the topic is not important, because gaps in knowledge must also be described in order to approach them (52). The review also clarifies that the included articles used the cross-sectional design to scientifically approach the health status of the health professional students. Such designs cannot generate reliable cause-and-effect findings, for example on the connection between studies and health (53). In addition, the descriptive results are not based on representative samples. In this respect, a general statement about the health situation of health professional students in Germany is not possible. Therefore, it makes sense to present and describe the main findings of each study in the results section of this scoping review. Additionally, the review found that most of the articles focused on nursing students. In terms of the healthcare system, this makes sense because the nursing professions are the largest occupational group within German’s healthcare (5). Against the background of the increasing interdisciplinary cooperation in daily healthcare practice (13), students of other health professions, such as physiotherapy, should also be taken into the health science research perspective. The female gender formed the largest sample in all included articles. In the German healthcare sector, the proportion of female professionals is 75% (54). The proportion of male students was underrepresented in the included studies. In the healthcare sector, the proportion of young male employees has risen from 19 to 25% (55). In addition, González and Peters (2021) found in their study that male students and young students drop out early (56). The start of a degree is a new and unknown phase of life, which young people are particularly challenged to cope with and can trigger a high level of subjective stress (19, 23, 28). In this respect, research into the subjective health of male students (57) and first-year students is also of great interest. After completing their studies, health professional students are entrusted with tasks that require additional skills, for example in relation to promoting the health literacy of patients (12). The included articles indicates that health professional students do not have sufficient health literacy themselves (47, 48). Students who are working alongside their studies more often reported a high subjective level of stress (43). Nursing studies in Germany are not financially rewarded (8). In this respect, many health professional students are encouraged to work alongside their demanding and highly structured studies (56). In particular, students with poor financial resources are burdened threefold by their studies, practical assignments and jobs. This stressful situation not only affects the subjective mental health of the students but also can lead to premature dropout from studying (56). These aspects should be viewed critically in relation to demographic change and the associated shortage of skilled workers (2, 9). Thus, health promotion and prevention in university setting is a particularly important public health topic (58). Our aim was not to produce a critically appraised and synthesized result to our research question. Due to this, an assessment of methodological limitations or risk of bias of the evidence included within a scoping review is generally not performed (38). For experts in the university environment as well as stakeholders in healthcare system, this scoping review offers a low-threshold insight into the health of the health professional students. However, further research is necessary on this target group to gather more knowledge about health situation. In summary, the Department of Nursing and Management at University of Applied Sciences Hamburg takes the scoping review as the starting point for further research projects.

### Limitations

The review has some limitations. As described in the protocol, the aims were not to provide a critical synthesis of the results related to the research questions. For this reason, the methodological limitations or risk of bias of the included articles not assessed. The focus of the scoping review was on health of professional students in German-speaking countries (Germany, Switzerland, Austria). As described in the introduction section, the professionalization of health professions in Germany still has a relatively recent development history (8, 13). Therefore, a limited number of articles on this topic were expected. Also, most articles focused on health of nursing students. The search strategy was iterative, and data extraction was conducted by one researcher due to limited resources. Finally, this scoping review was an enormous research effort, so our results are from the 2012 state.

### Conclusion

This scoping review highlighted the current scope of research regarding the health of health professional students in German-speaking countries. The discussion of challenges and problems as well as future important challenges offered valuable insights for the research community and approaches for health promotion interventions in university settings. It should be noted that existing research in this field is still limited, and research results regarding the health situation of health professional students in Germany is not representative. Therefore, more research is needed to generate knowledge and describe the health situation of this specific group of students in a comprehensive way. Future studies should aim to collect nationwide representative data on the health of health professional students. By using a longitudinal study design, valuable insights can be gained into the impact of study demands on students’ health. Such studies are important for developing targeted interventions that address the association between health and study. In addition, the use of participatory research designs is advised to assess and address students’ subjective perspectives on their health.

## Author contributions

I-NJ designed the scoping review and developed the initial draft of the manuscript. AMN supported evidence selection, data extraction and data verify several times. CP-E is the project leader and the advisor of the dissertation. CP-E, AMN, and PK critically reviewed the manuscript, and provided inputs for improvement. All authors contributed to the article and approved the submitted version.

## Conflict of interest

The authors declare that the research was conducted in the absence of any commercial or financial relationships that could be construed as a potential conflict of interest.

## Publisher’s note

All claims expressed in this article are solely those of the authors and do not necessarily represent those of their affiliated organizations, or those of the publisher, the editors and the reviewers. Any product that may be evaluated in this article, or claim that may be made by its manufacturer, is not guaranteed or endorsed by the publisher.

## Supplementary material

The Supplementary material for this article can be found online at: https://www.frontiersin.org/articles/10.3389/fpubh.2023.1243324/full#supplementary-material
