# Supplementary material for: Health of (dual) health professional students in German-speaking countries: a scoping review
Source: Front Public Health. 2023 Sep 19;11:1243324. doi: 10.3389/fpubh.2023.1243324 (PMC10546053; doi:10.3389/fpubh.2023.1243324)
Supplement: Supplementary file 1 [file Table_1.pdf]

Appendix 1
Full search strategies/terms electronic databases (n=11)

MEDLINE (via PubMed)
Search date: February 20, 2022

#	Query	Results
**1**	″Students, Health Occupations″ [Mesh] OR ″health profession* student*″ OR ″healthcare student*″ OR ″health-care student*″ OR ″health care student*″ OR "academic health profession*" OR "dual student*" OR "nursing student*" OR "physiotherapy student*" OR "physical therapy student*" OR "occupational therapy student*" OR "speech therapy student*" OR "allied health student*"	126.652
**2**	health OR healthy	6.893.179
**3**	"Subjective health status" OR "health related quality of life" OR “quality of life” OR “well-being” OR "health perception" OR "perceived health" OR “health-related lifestyle factors” OR "health problem*" OR “health promotion” OR "physical health" OR "physical health problem*" OR “physical inactivity” OR "physical activity" OR “mental well-being” OR "mental health" OR "mental health problem*" OR "tobacco use" OR "substance* use" OR smok* OR cigarette OR alcohol OR “alcohol consumption” OR drug OR "eating behavio*" OR "nutritional habit*" OR “body mass index” OR “body weight” OR overweight OR obese OR “health-related behavio*" OR “risk health behavio*" OR “health behavior” OR “unhealthy behavio*” OR medication OR “health literacy” OR “self-efficacy” OR stress* OR burden OR “stress level” OR “academic requirements”	14.629.733
**4**	German* OR "german*-speaking region" OR "german*-speaking area" OR switz* OR austria	2.710.501
**5**	#1 AND #2 AND #3 AND #4	1.936
**6**	#1 AND #2 AND #3 AND #4<br>(Filters: Abstract, last 10 years, Humans, English, German, Adult: 19+ years)	529

Records identified: 529. Inclusion: 5 (EndNote)

CINAHL (via EBSCO)
Search date: February 22, 2023

#	Query	Results
**1**	(″Students, Health Occupations″ OR ″health profession* student*″ OR ″healthcare student*″ OR ″health-care student*″ OR ″health care student*″ OR "academic health profession*" OR "dual student*" OR "nursing student*" OR "physiotherapy student*" OR "physical therapy student*" OR "occupational therapy student*" OR "speech therapy student*" OR "allied health student*") **AND** ("Subjective health status" OR "health related quality of life" OR “quality of life” OR “well-being” OR "health perception" OR "perceived health" OR “health-related lifestyle factors” OR "health problem*" OR “health promotion” OR "physical health" OR "physical health problem*" OR “physical inactivity” OR "physical activity" OR “mental well-being” OR "mental health" OR "mental health problem*" OR "tobacco use" OR "substance* use" OR smok* OR cigarette OR alcohol OR “alcohol consumption” OR drug OR "eating behavio*" OR "nutritional habit*" OR “body mass index” OR “body weight” OR overweight OR obese OR “health-related behavio*" OR “risk health behavio*" OR “health behavior” OR “unhealthy behavio*” OR medication OR “health literacy” OR “self-efficacy” OR stress* OR burden OR “stress level” OR “academic requirements”) **AND** (German* OR "german*-speaking region" OR "german*-speaking area" OR switz* OR austria)<br>**Limiters:** Abstract Available<br>**Search modes:** Find all my search terms	143
**2**	(″Students, Health Occupations″ OR ″health profession* student*″ OR ″healthcare student*″ OR ″health-care student*″ OR ″health care student*″ OR "academic health profession*" OR "dual student*" OR "nursing student*" OR "physiotherapy student*" OR "physical therapy student*" OR "occupational therapy student*" OR "speech therapy student*" OR "allied health student*") **AND** ("Subjective health status" OR "health related quality of life" OR “quality of life” OR “well-being” OR "health perception" OR "perceived health" OR “health-related lifestyle factors” OR "health problem*" OR “health promotion” OR "physical health" OR "physical health problem*" OR “physical inactivity” OR "physical activity" OR “mental well-being” OR "mental health" OR "mental health problem*" OR "tobacco use" OR "substance* use" OR smok* OR cigarette OR alcohol OR “alcohol consumption” OR drug OR "eating behavio*" OR "nutritional habit*" OR “body mass index” OR “body weight” OR overweight OR obese OR “health-related behavio*" OR “risk health behavio*" OR “health behavior” OR “unhealthy behavio*” OR medication OR “health literacy” OR “self-efficacy” OR stress* OR burden OR “stress level” OR “academic requirements”) **AND** (German* OR "german*-speaking region" OR "german*-speaking area" OR switz* OR austria)	
**a**	**Limiters:** Abstract Available; Published Date: 20120101-20231231<br>**Search modes:** Find all my search terms	108
**b**	**Limiters:** Abstract Available; Published Date: 20120101-20231231<br>**Search modes:** Boolean/Phrase	38
**3**	(″Students, Health Occupations″ OR ″health profession* student*″ OR ″healthcare student*″ OR ″health-care student*″ OR ″health care student*″ OR "academic health profession*" OR "dual student*" OR "nursing student*" OR "physiotherapy student*" OR "physical therapy student*" OR "occupational therapy student*" OR "speech therapy student*" OR "allied health student*") **AND** (health or healthy) **AND** (germany or german or deutschland or deutsch)<br>**Limiters:** Abstract Available<br>**Search modes:** Boolean/Phrase	61

**4**	(″Students, Health Occupations″ OR ″health profession* student*″ OR ″healthcare student*″ OR ″health-care student*″ OR ″health care student*″ OR "academic health profession*" OR "dual student*" OR "nursing student*" OR "physiotherapy student*" OR "physical therapy student*" OR "occupational therapy student*" OR "speech therapy student*" OR "allied health student*") **AND** (health or healthy) **AND** (germany or german or deutschland or deutsch)<br>**Limiters:** Abstract Available; Published Date: 20120101-20231231<br>**Search modes:** Find all my search terms	158
		470

Records identified: 470. Inclusion: 13 (EndNote)

CareLit (German-language journal literature for nursing and health professions)
Search date: February 23, 2023

#	Query	Results
**1**	studierende AND gesundheit AND deutschland<br>**Suchkriterien**: Ab 2012-2023	1.341
**2**	studierende AND gesundheit AND gesundheitsverhalten AND gesundheitsförderung UND deutschland<br>**Suchkriterien:** ab 2012-2023	261
**3**	studierende AND gesundheitsfachberufe AND (gesundheit OR gesundheitsverhalten) AND dual	116
		1.718

Records identified: 1.718. Inclusion: 5 (EndNote).

LIVIVO
Search date: March 1, 2023

#	Query	Results
**1**	(″Students, Health Occupations″ OR ″health profession* student*″ OR ″healthcare student*″ OR ″health-care student*″ OR ″health care student*″ OR "academic health profession*" OR "dual student*" OR "nursing student*" OR "physiotherapy student*" OR "physical therapy student*" OR "occupational therapy student*" OR "speech therapy student*" OR "allied health student*") **AND** ("Subjective health status" OR "health related quality of life" OR "quality of life" OR "well-being" OR "health perception" OR "perceived health" OR "health-related lifestyle factors" OR "health problem*" OR "health promotion" OR "physical health" OR "physical health problem*" OR "physical inactivity" OR "physical activity" OR "mental well-being" OR "mental health" OR "mental health problem*" OR "tobacco use" OR "substance* use" OR smok* OR cigarette OR alcohol OR "alcohol consumption" OR drug OR "eating behavio*" OR "nutritional habit*" OR "body mass index" OR "body weight" OR overweight OR obese OR "health-related behavio*" OR "risk health behavio*" OR "health behavior" OR "unhealthy behavio*" OR medication OR "health literacy" OR "self-efficacy" OR stress* OR burden OR "stress level" OR "academic requirements") **AND** (German* OR "german*-speaking region" OR "german*-speaking area" OR switz* OR austria)<br>**Limiters:** -/-<br>**Search modes:** Open search	48
**2**	(″Students, Health Occupations″ OR ″health profession* student*″ OR ″healthcare student*″ OR ″health-care student*″ OR ″health care student*″ OR "academic health profession*" OR "dual student*" OR "nursing student*" OR "physiotherapy student*" OR "physical therapy student*" OR "occupational therapy student*" OR "speech therapy student*" OR "allied health student*") **AND** ("Subjective health status" OR "health related quality of life" OR "quality of life" OR "well-being" OR "health perception" OR "perceived health" OR "health-related lifestyle factors" OR "health problem*" OR "health promotion" OR "physical health" OR "physical health problem*" OR "physical inactivity" OR "physical activity" OR "mental well-being" OR "mental health" OR "mental health problem*" OR "tobacco use" OR "substance* use" OR smok* OR cigarette OR alcohol OR "alcohol consumption" OR drug OR "eating behavio*" OR "nutritional habit*" OR "body mass index" OR "body weight" OR overweight OR obese OR "health-related behavio*" OR "risk health behavio*" OR "health behavior" OR "unhealthy behavio*" OR medication OR "health literacy" OR "self-efficacy" OR stress* OR burden OR "stress level" OR "academic requirements") **AND** (German* OR "german*-speaking region" OR "german*-speaking area" OR switz* OR austria)<br>**Limiters:** 2012-2023<br>**Search modes:** Open search	33
		81

Records identified: 81. Inclusion: 2 (EndNote)

Scopus
Search date: March 8, 2023

#	Query	Results
**1**	(TITLE-ABS-KEY ("health profession* student*" OR "nursing student*" OR "occupational student*" OR "physiotherapy student*" OR "speech-therapy student*") AND TITLE-ABS-KEY (health OR "health status" OR "health beahvior*" OR "mental health" OR "physical health" OR smoking OR "tobacco use" OR "eating behavior*") AND TITLE-ABS-KEY ("german*" OR "german-speaking countrie*" OR switz* OR austria))	139
**2**	(TITLE-ABS-KEY ("nursing student*" OR "occupational student*" OR "physiotherapy student*" OR "speech-therapy student*") AND TITLE-ABS-KEY ("health behavior*" OR "health habit*" OR "health promotion") AND TITLE-ABS-KEY ("german*" OR switz* OR austria OR "university setting*"))	12
**3**	(title-abs-key("health*") AND title-abs-key(student*) AND title-abs-key(german*)) AND (exclude (subjarea,"medi") OR exclude (subjarea,"soci") OR exclude (subjarea,"psyc") OR exclude (subjarea,"envi") OR exclude (subjarea,"bioc") OR exclude (subjarea,"arts") OR exclude (subjarea,"neur") OR exclude (subjarea,"agri")) AND (exclude (subjarea,"mult") OR exclude (subjarea,"dent") OR exclude (subjarea,"comp") OR exclude (subjarea,"engi") OR exclude (subjarea,"vete") OR exclude (subjarea,"math") OR exclude (subjarea,"busi") OR exclude (subjarea,"phar") or exclude (subjarea,"econ")) and (exclude (subjarea,"phys") OR exclude (subjarea,"eart") OR exclude (subjarea,"ener") OR exclude (subjarea,"immu") OR exclude (subjarea,"chem") OR exclude (subjarea,"mate") OR exclude (subjarea,"undefined"))	39
**4**	(title-abs-key ("student*" OR "dual student*" OR "dual-student*") AND title-abs-key (health OR healthy* OR "health promotion") AND title-abs-key (german*)) AND (exclude (subjarea , "medi") OR exclude (subjarea , "soci") OR exclude (subjarea , "psyc") OR exclude (subjarea , "envi") OR exclude (subjarea , "bioc") OR exclude (subjarea , "arts") OR exclude (subjarea , "neur") OR exclude (subjarea , "agri")) AND (exclude (subjarea , "mult") OR exclude (subjarea , "dent") OR exclude (subjarea , "comp") OR exclude (subjarea , "engi") or exclude (subjarea , "vete") or exclude (subjarea , "busi") OR exclude (subjarea , "phar") OR exclude (subjarea , "econ")) AND (exclude (subjarea , "phys") OR exclude (subjarea , "eart") OR exclude (subjarea , "ener") OR exclude (subjarea , "immu") OR exclude (subjarea , "chem") OR exclude (subjarea , "mate") OR exclude (subjarea , "math") OR exclude (subjarea , "undefined"))	38
		228

Records identified: 228. Inclusion: 4 (EndNote).

PSYNDEX (via Ovid)
Search date: March 2, 2023

#	Query	Results
**1**	(health profession* student* OR healthcare student* OR health-care student* OR health care student* OR academic health profession* OR dual student* OR nursing student* OR physiotherapy student* OR physical therapy student* OR occupational therapy student* OR speech therapy student* OR allied health student*) AND (health professional* OR healthcare) AND (health* OR healthy) AND (german* OR switz* OR austria)<br>**Limiters: 2012-2023**	712
**2**	student* AND healthcare* AND (health* OR healthy) AND german*<br>**Limiters: 2012-2023**	471
**3**	student* AND health professional* AND (health* OR healthy) AND german*<br>**Limiters: 2012-2023**	232
		1.415

Records identified: 1.415. Inclusion: 6 (EndNote)

PEDro
Search date: March 4, 2023

#	Query	Results
**1**	student* health promotion* intervention*	144

Records identified: 144. Inclusion: 0 (EndNote)

OTseeker
Search date: March 7, 2023

#	Query	Results
**1**	student* and health* (any fields)	82
**2**	student* and health* (titel/abstract)	6
**3**	dual student* and health* (titel/abstract)	0
**4**	"occupational therapy student*"	6
		94

Records identified: 94. Inclusion: 0 (EndNote)

OpenGrey (University London)
Search date: March 11, 2023

#	Query	Results
**1**	("student* health professional*" OR "student* healthcare*" OR "student* health care") AND (health OR healthy OR "health behavior*") AND (german* OR switz* OR austria)	14
**2**	"health professional student*" and health and (german* or switz* or austria)	26
**3**	("nursing student*" OR "occupational student*" OR "physiotherapy student*" OR "speech-therapy student*") AND (health OR "health status" OR "health behavior" OR "health promotion") AND (german* OR switz* OR austria)<br>**Limiters**: Publication Date: 01.01.2012-13.03.2023; Discipline: nursing, physical therapy, occupational therapy & rehabilitation	392
		432

Records identified: 432. Inclusion: 0 (EndNote)

Google Scholar
Search date: March 14, 2023 / March 15, 2023

Search machine	Results
**Google Scholar:**<br>**#1** (studierende UND pflegeberuf UND therapieberuf UND deutschland UND gesundheit UND hochschule)<br>**Zeitraum:** 2012-2023	148
**#2** studierende UND (pflege ODER therapie) UND (deutschland ODER schweiz ODER österreich) UND gesundheit UND gesundheitsförderung UND hochschule<br>**Zeitraum:** 2012-2023	839
**#3** studierende UND pflegeberuf UND therapieberuf UND deutschland UND gesundheitsverhalten UND hochschule<br>**Zeitraum:** 2012-2023	15
**#4** studierende UND gesundheitsfachberuf UND (gesundheit OR gesundheitskompetenz) UND deutschland<br>**Zeitraum:** 2012-2023	65
**#5** studierende UND gesundheit UND hochschule UND deutschland<br>**Zeitraum:** 2012-2023	16.800

Records identified:17.867. Inclusion: 7 (EndNote)

Online library catalog (Hamburg University of Applied Sciences)
Search date: March 16, 2023

#	Query	Results
**1**	"health profession student*" AND (german* OR switz* OR austria)<br>**Limiters**: Date: 2012-2023	20
**2**	("health profession student*" OR "nursing student*" OR "occupational student*" OR "physiotherapy student*" OR "speech-therapy student*") AND (German* OR switz* OR austria) AND (health OR healthy OR "health behavior")<br>**Limiters:** Date: 2012-2023	56
**3**	("nursing student*" OR "occupational student*" OR "physiotherapy student*" OR "speech-therapy student*") AND (German* OR switz* OR austria) AND (health OR healthy OR "health behavior")<br>**Limiters:** Date: 2012-2023	24
**4**	"nursing student*" AND (health OR healthy OR "health behavior")<br>**Limiters:** Date: 2012-2023	860
		960

Records identified: 960. Inclusion: 0 (EndNote)
